# Assessment of the ability, perception, and readiness of nurses to manage tracheostomy-related complications in Riyadh City: a cross-sectional study

**DOI:** 10.1186/s12912-022-01101-y

**Published:** 2022-11-23

**Authors:** Fahad Zarraq Alotaibi, Reema Alkhatabi, Lujain Allowaihiq, Hassan Alhazzani, Ghada Alshehri, Eman A. Hajr, Feras Alkholaiwi

**Affiliations:** 1 College of Medicine, Imam Mohammed Ibn Saud Islamic University, Othman Bin Affan Road Alnada-7544, 13317-4233 Riyadh, Kingdom of Saudi Arabia; 2 Department of Otorhinolaryngology-Head and Neck Surgery, Imam Mohammad Ibn Saud Islamic University, Othman Bin Affan Road Alnada-7544, 13317-4233 Riyadh, Kingdom of Saudi Arabia; 3 College of Medicine, King Saud University, King Khalid Road 11451, Riyadh, 2454-1145 Saudi Arabia

**Keywords:** Tracheostomy, Acute Respiratory Failure, Nurses, Knowledge, Complications, Otolaryngology, Mechanical Ventilation

## Abstract

**Background:**

Nursing staff play a key role in the bedside management of tracheostomized patients in both intensive care units and general floors. Effective provision of postoperative care to these patients by nurses is critical. This study aimed to assess the readiness of nursing staff to manage tracheostomy-related complications in Riyadh, Saudi Arabia.

**Methods:**

This cross-sectional study enrolled 395 nurses in Riyadh, Saudi Arabia. A self-administered questionnaire developed by the authors was distributed to nurses via an online survey. The questionnaire assessed nurses’ socio-demographic characteristics and their knowledge and practices regarding tracheostomy indications, postoperative care, and management of its complications.

**Results:**

Of respondents, approximately 59% (232/395) had poor knowledge regarding tracheostomy-related complications and indications, 39% (154/395) had moderate knowledge, and only 2%(9/395) had good knowledge. Several factors were associated with increased knowledge, including having a higher education degree, working in a tertiary/quaternary hospital, being a government employee, caring for tracheostomized patients, and attending courses or lectures on managing tracheostomized patients.

**Conclusions:**

Knowledge level and practices regarding tracheostomy-related complications and indications among nurses remain limited. In-service continuous professional development programs for nurses working in all hospital settings are strongly recommended. Further research is warranted to elicit the major factors contributing to inadequate knowledge and practices among nurses in Riyadh.

**Supplementary Information:**

The online version contains supplementary material available at 10.1186/s12912-022-01101-y.

## Background

Tracheostomy, one of the oldest known surgical procedures, is indicated when the airway is compromised and long-term ventilation is required [1]. Tracheostomy-related adverse events constitute a significant global issue, accounting for half of all airway-related deaths and hypoxic brain damage cases in critical care units [2]. Generally, these complications and adverse events are related to poor tracheostomy care and practices [3]. Reports from the United Kingdom (UK) have identified that adverse events occur in 20–30% of all hospitalized patients managed with a tracheostomy [4].

Tracheostomy care and management are key skills required by all nurses, as nurses are considered crucial members of the tracheostomy care team regardless of the nurse-designated unit of practice [5], especially in situations of bed management crisis. During the pandemic, where the care of tracheostomized patients was not limited to intensive care, high dependency, or otolaryngology floors, these patients could be admitted and managed on any hospital floor.

Patients with tracheostomy have a high risk of airway obstruction, hemorrhage, and local and systemic infection. Poor knowledge and practices regarding tracheostomy care by bedside nurses can lead to complications that are life-threatening to the patient. Nurses play a major role in promoting positive outcomes in patients with tracheostomy, as nurses spend more time with patients receiving tracheostomy care. Nevertheless, the knowledge and practices of nurses regarding tracheostomy care have been reported to be very poor, thus negatively impacting patient outcomes [6]. As research on this topic in Saudi Arabia is limited, this study aimed to evaluate the current level of knowledge and ability of nurses in Saudi Arabia to manage tracheostomy complications.

## Methods

### Study design

This descriptive cross-sectional study was performed to assess the ability and knowledge of nurses in Riyadh city, Saudi Arabia regarding the management of tracheostomy-related complications.

### Study participants and sample size

#### Inclusion criteria

Study participants were nurses from different departments of Riyadh hospitals, including both adult and pediatric sections.

#### Exclusion criteria

Nurses practicing outside Riyadh city, nursing students, interns, and other health care providers were excluded from the study.

#### Ethical consideration

This study was reviewed and approved by the ethical research committee of the Institutional Review Board at Imam Mohammed Ibn Saud Islamic University, Riyadh, Saudi Arabia (HAPO-01-R-0011, Project No. 118–2021). All methods were performed in accordance with the relevant guidelines and regulations. The responses to the online survey were anonymous, with no tracking of e-mail addresses or identifying information. Electronic informed consent was obtained from all the participants—a consent statement was present at the beginning of the survey, with the ‘Yes’ option allowing the participant to continue. Participation was voluntary, with the option to withdraw at any time.

#### Sampling procedure

The sample size was calculated using a Krejcie and Morgan table to determine the appropriate sample size [7]. Based on the total number of 75,000 practicing nurses in Riyadh in 2018 [8], the estimated population size for this study was 382.

### Data collection procedure

An electronic questionnaire designed by the authors was created on Google forms and sent as an email to randomly selected registered nurses from the nursing staff database in the Saudi commission for health specialties, which includes all registered nurses licensed to practice in Saudi Arabia. The study background and purpose were explained in the email. Of a total of 500 nurses, 395 responded, with a response rate of 79%. Data were collected from September 2021 to February 2022.

#### Data collection instrument

As the data collection tools used in previous studies failed to address our objectives, the senior authors (FA and EH) created a survey to collect data for this study. Questions were designed after thoroughly reviewing the current literature and guidelines on the subject [9–13]. The tool was administered to clinical experts (tracheostomy team nurses, intensive care physicians, otolaryngologists) and academic experts to assess the suitability of all content to be measured. The content validity ratio of the instrument was considered in this study after being rated by experts using the content validity ratio formula as follows: (CVR) = [(E – (N/2))/(N/2)], where (N) and (E) stand for the total number of experts and number of experts who rated the instrument as essential, respectively. The CVR from the instrument of this study was 0.85, which was closer to 1.0; hence, the instrument was considered valid.

The final version of the questionnaire comprised 32 questions divided into the following four domains: (1) demographic data of participants (4 questions), (2) practice settings and experience (4 questions), (3) basic knowledge of tracheostomy tube components and indications (13 questions), and (4) management of tracheostomy complications (11 questions) (Supplementary Material, Additional File 1).

The knowledge of nurses regarding managing tracheostomy-related complications was assessed using their responses to 21 of the 33 questions (13 and 8 questions from the third and fourth domains, respectively), whereby correct answers were identified and coded with 1, and incorrect answers were coded with 0. All questions were single-answer except for six questions with multiple correct answers, including questions 1 (10 correct answers), 9 (4 correct answers), 14 (3 correct answers), 18 (5 correct answers), 20 (6 correct answers), and 21 (3 correct answers). The Knowledge Questionnaire comprised 49 points representing all correct answers. A total knowledge score ranging from 1 to 49 points was generated, with a higher score indicating greater knowledge of managing tracheostomy-related complications.

### Data analysis

Data were analyzed using SPSS version 26 (IBM Corp, Armonk, NY, USA). Demographic characteristics, practice settings, basic knowledge, and management of complications were analyzed using descriptive statistics with numbers, percentages, and means ± standard deviations. The differences in knowledge scores according to the socio-demographic characteristics of participants were calculated using the Mann–Whitney Z-test and Kruskal–Wallis H-test. Additionally, normality was examined using the Shapiro–Wilk and Kolmogorov–Smirnov tests. As knowledge scores were non-normally distributed, nonparametric tests were performed. Statistical significance was set at *p* < 0.05.

To determine the knowledge level of nurses, we generated a score percentage for each nurse (points achieved/49 total points × 100) and categorized them as follows: > 75%, good knowledge; 50–75%, average or moderate knowledge; and < 50%, poor knowledge regarding the management of tracheostomy-related complications.

## Results

**Fig. 1:**
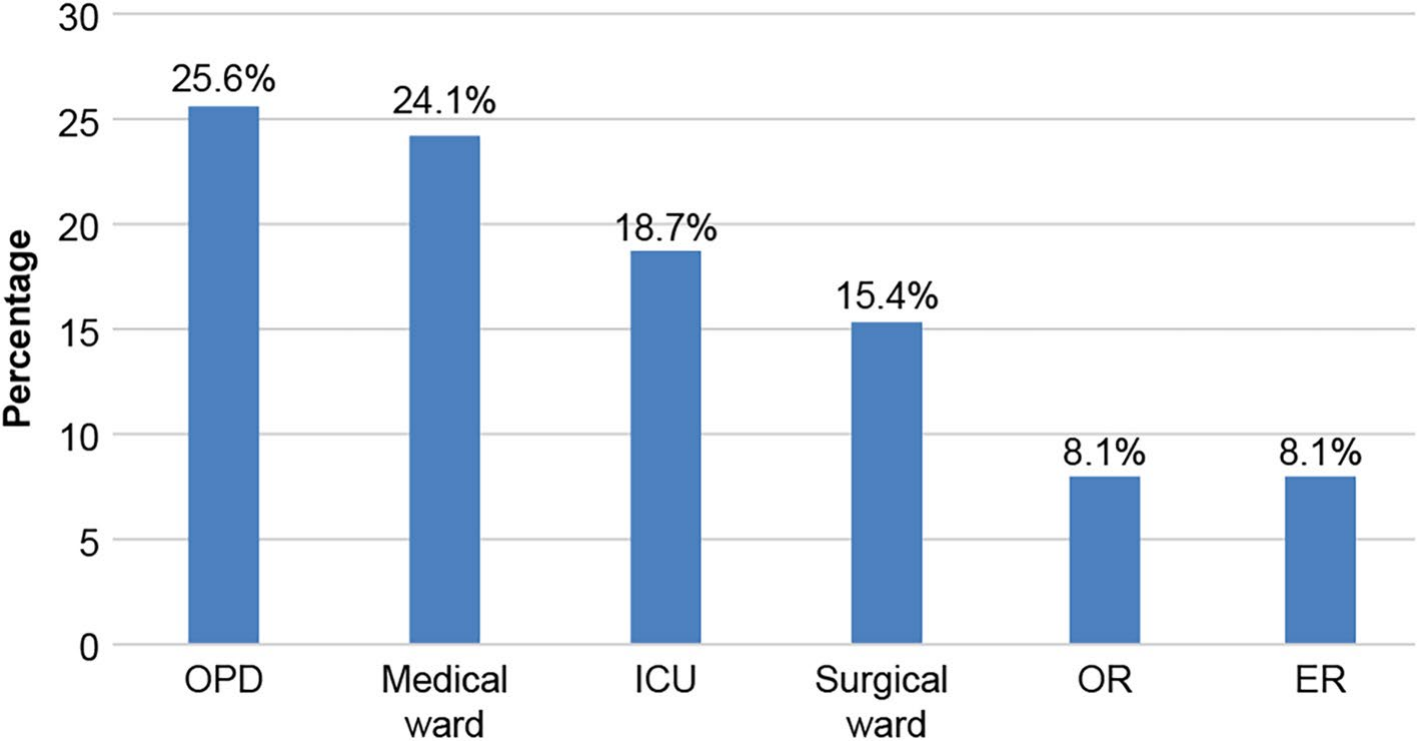
Distribution of nurses’ workplaces. The most common workplace is the outpatient department (OPD) (25.6%), followed by the medical ward (24.1%) and intensive care unit (ICU) (18.7%)

**Fig. 2:**
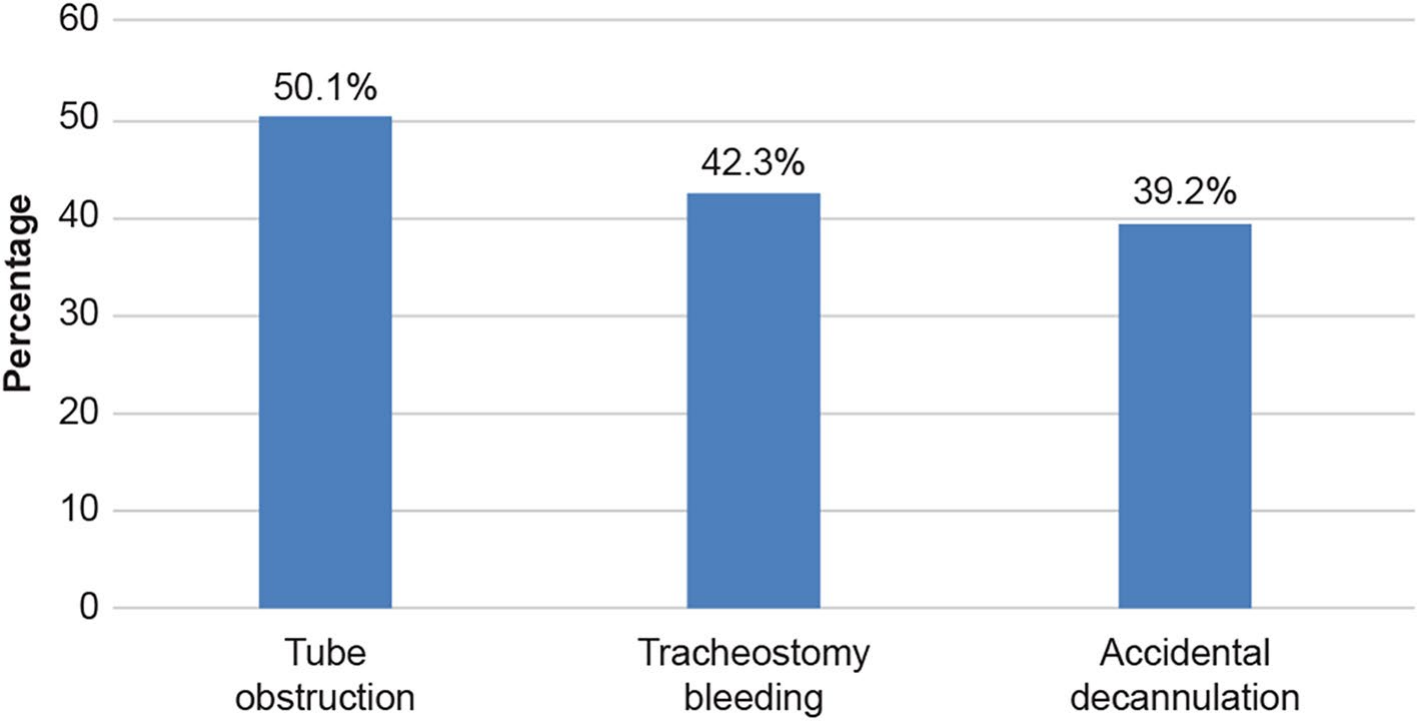
Previous experience with complications. Among nurses, 50.1% had encountered tube obstruction, 42.3% had experience with tracheostomy bleeding, and 39.2% had dealt with accidental decannulation

In total, 395 nurses responded to the survey. Table 1 outlines the socio-demographic characteristics of nurses: 46.8% were aged between 31 and 40 years, with a predominance of female individuals (82%). Most nurses held bachelor’s degrees (77.5%), with 42.5% having 5-10 years of work experience. Nearly half of nurses were employed in tertiary hospitals (57.7%). Most of the respondents worked in government institutions (85.6%). Almost 25% of the nurses worked in inpatient medical wards (Fig. 1). Of nurses, 78.5% had cared for patients with tracheostomies, and > 50% had dealt with tracheostomy complications before (Fig. 2). Approximately 35.2% of nurses had attended lectures or courses related to managing patients with tracheostomies.

**Table 1:** Socio-demographic characteristics of nurses (*n* = 395)

Study variables	n (%)
Age group	
20–30 years	151 (38.2%)
31–40 years	185 (46.8%)
> 40 years	59 (14.9%)
Sex	
Male	71 (18.0%)
Female	324 (82.0%)
Education level	
Diploma	72 (18.2%)
Bachelor’s degree	306 (77.5%)
Master’s degree or PhD	17 (04.3%)
Years in practice	
< 5 years	112 (28.4%)
5–10 years	168 (42.5%)
> 10 years	115 (29.1%)
Level of care provided by hospital	
Primary	96 (24.3%)
Secondary	71 (18.0%)
Tertiary	228 (57.7%)
Type of institution	
Government	338 (85.6%)
Private	57 (14.4%)
Have you taken care of tracheostomized patients before?	
Yes	310 (78.5%)
No	85 (21.5%)
Have you ever taken a course or lecture about managing patients with tracheostomy?	
Yes	256 (64.8%)
No	139 (35.2%)

**Fig. 3:**
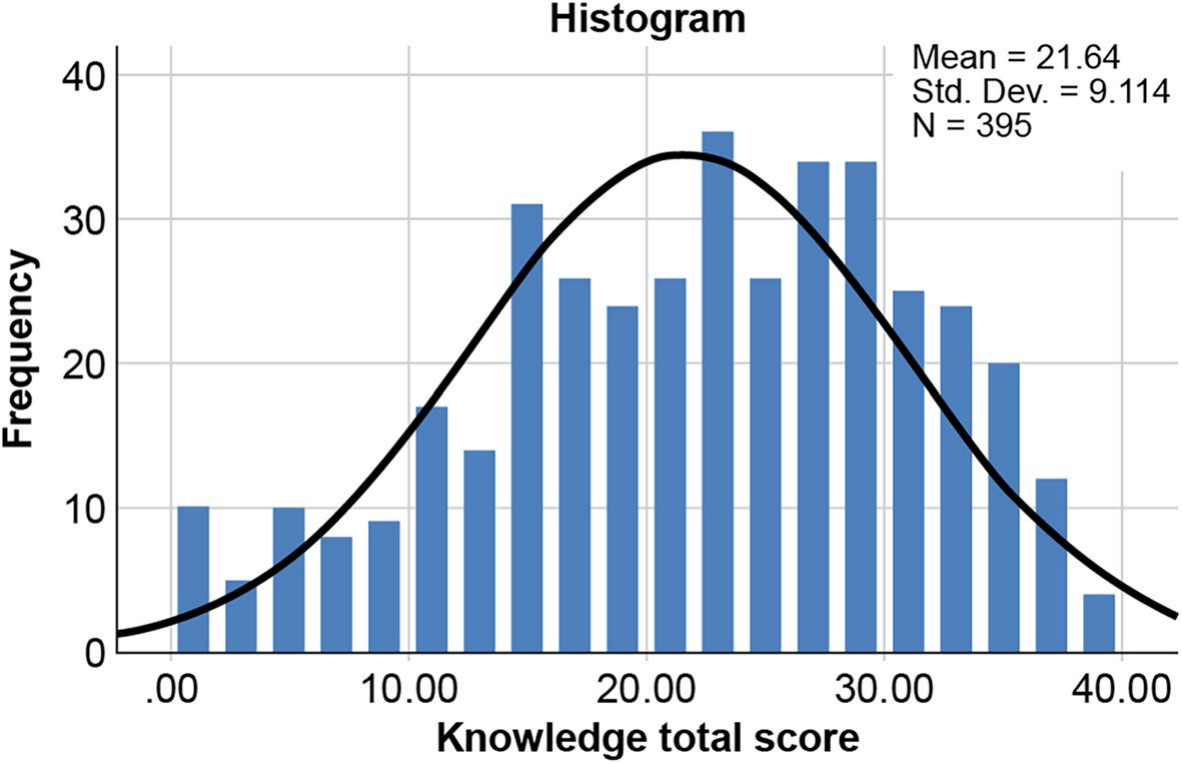
Histogram of knowledge scores. As knowledge scores were non-normally distributed, non-parametric tests were applied for statistical analysis (*p* < 0.05)

**Fig. 4:**
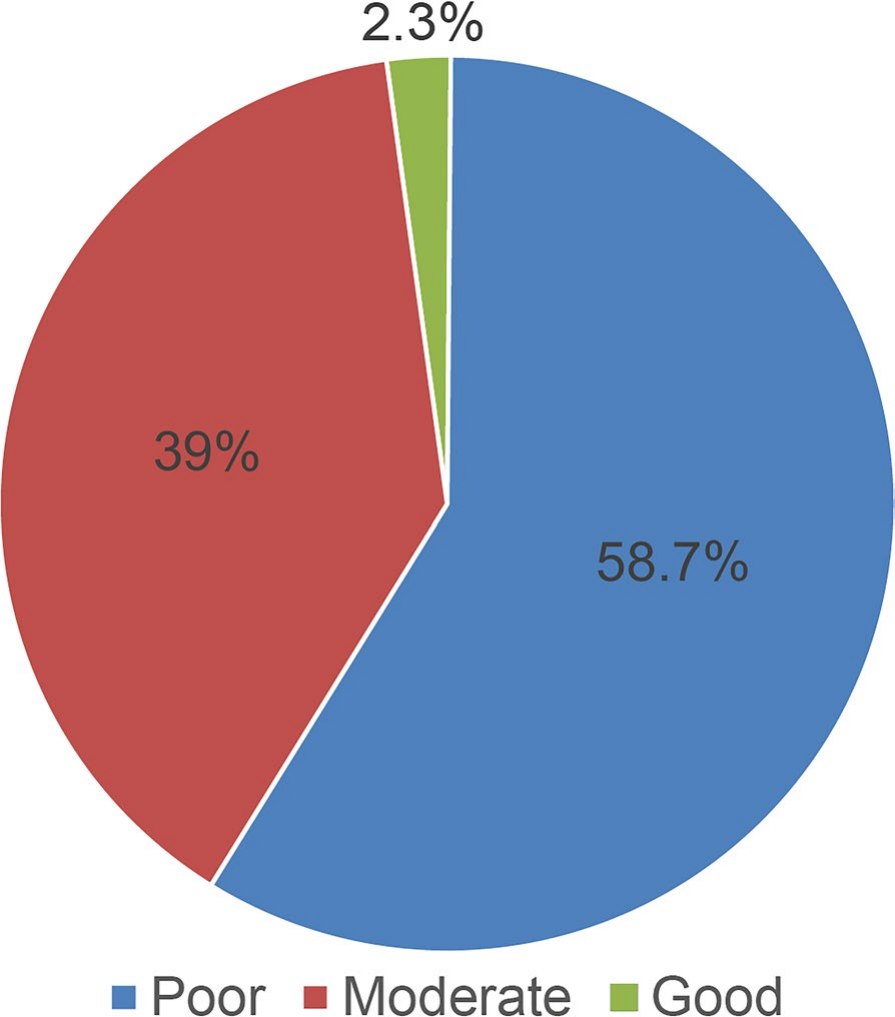
Level of knowledge. The pie chart depicts the overall level of knowledge among the study participants, with 58.7% (*n* = 232), 39% (*n* = 154), and 2.3% (*n* = 9) having poor, moderate, and good knowledge scores, respectively

Results of the assessment of knowledge on managing tracheostomy-related complications are presented in Table 2. Most nurses thought that the main reason for performing tracheostomies was airway obstruction (82%), followed by prolonged mechanical ventilation (68.1%) and inability to intubate (58.5%). Of nurses, 40.3% correctly stated that the inner cannula should be checked and cleaned twice daily. However, nurses recognized that the ideal characteristics of a tracheostomy stoma were clean and dry, while the ideal cuff pressure was < 25 cm (45.6%). Approximately 48.6% of the respondents knew that the cuff should be deflated when removing the patient from a mechanical ventilator. Of respondents, 76.7% believed that patients with tracheostomy required “humidification.” Of nurses, 47.3% knew that suctioning should only be performed when necessary, and 80.5% knew that audible or visible signs of secretion in the tube were the most common indications for suctioning. Most respondents correctly identified the appropriate suction pressure as 80–120 mmHg (59.2%). Only 33.2% of respondents knew that the proper suction tube length relative to the cannula length was the same as that of the suction tube. Furthermore, there was a lack of knowledge regarding when to change the first tube, with only 36.2% indicating a response of 5-10 days. A tracheal dilator was correctly selected as the piece of bedside tracheostomy equipment that should be kept at all times (62.3%). Only 21.3% of the respondents knew that reinserting the tube should be the first step after accidental decannulation. In total, 32.9% of participants reported that inserting suction was the first measure of tube obstruction. Oxygen saturation was correctly selected as a sign of tube obstruction (71.6%), followed by breathing difficulty (67.6%). Fever was correctly selected as a sign of infection (92.7%), followed by foul odor mucus (63,8%) and increased respiratory rate (46.6%), whereas systemic antibodies were wrongly selected as a measure to reduce tracheostomy-related infections (69.1%). Collectively, the mean ± standard deviation score was 21.6 ± 9.11, with poor, moderate, and good knowledge detected in 58.7%, 39%, and 2.3% of the nurses, respectively (Figs. 3 and 4).

**Table 2:** Assessment of knowledge on managing tracheostomy-related complications (*n* = 348)

Knowledge statement	N (%)
1. Which of the following is an indication to perform a tracheostomy?^a^	
Airway obstruction^b^	324 (82.0%)
Prolonged mechanical ventilation^b^	269 (68.1%)
Inability to intubate^b^	231 (58.5%)
Neck trauma^b^	175 (44.3%)
Facial fracture^b^	159 (40.3%)
Neuromuscular disease^b^	156 (39.5%)
Bilateral vocal cord paralysis^b^	147 (37.2%)
Infections	141 (35.7%)
Congenital anomaly^b^	125 (31.6%)
Neoplasm^b^	87 (22.0%)
Prophylaxis in head and neck procedures^b^	78 (19.7%)
2. How frequently should the inner cannula be checked and cleaned?	
Once a day	139 (35.2%)
Twice a day^b^	159 (40.3%)
Every 2 days	33 (08.4%)
Once a week	18 (04.6%)
I do not know	46 (11.6%)
3. Which of the following is a component of the tracheostomy care kit?	
Inner cannula	177 (44.8%)
Obturator	49 (12.4%)
Plug	29 (07.3%)
Hydrogen peroxide^b^	68 (17.2%)
I do not know	72 (18.2%)
4. What are the characteristics of an ideal tracheostomy stoma?	
Clean and dry^b^	333 (84.3%)
Wet	18 (04.6%)
Full of granulation tissues	13 (03.3%)
I do not know	31 (07.8%)
5. What is the ideal cuff pressure?	
Below 25 cm H2O^b^	180 (45.6%)
Above 26 cm H2O	42 (10.6%)
Below 35 cm H2O	06 (01.5%)
Above 35 cm H2O	33 (08.4%)
I do not know	134 (33.9%)
6. When should the cuff be deflated?	
When the patient is off MV^b^	192 (48.6%)
Cuff should always be kept inflated	123 (31.1%)
I do not know	80 (20.3%)
7. Do patients with a tracheostomy require ‘humidification’?	
Yes^b^	303 (76.7%)
No	46 (11.6%)
I do not know	46 (11.6%)
8. How frequent should ‘suctioning’ be performed?	
Only when indicated^b^	187 (47.3%)
Routinely every 2 h	101 (25.6%)
Routinely every 4 h	40 (10.1%)
Routinely on each shift	32 (08.1%)
I do not know	35 (08.9%)
9. Which of the following is an indication for suctioning?^a^	
Audible or visual signs of secretions in the tube*	318 (80.5%)
Signs of respiratory distress^b^	274 (69.4%)
Blocked or partially blocked tube^b^	265 (67.1%)
Vomiting	138 (34.9%)
Desaturation on pulse oximetry^b^	224 (56.7%)
I do not know	30 (07.6%)
10. What is the appropriate suctioning pressure?	
50-70 mmHg	34 (08.6%)
80-120 mmHg^b^	234 (59.2%)
120-150 mmHg	38 (09.6%)
I do not know	89 (22.5%)
11. Appropriate length of the suction tube in relation to cannula length	
Shorter	45 (11.4%)
Same length^b^	131 (33.2%)
Longer	157 (39.7%)
I do not know	62 (15.7%)
12. When should the sutures be removed after surgical insertion?	
5-7 days^b^	170 (43.0%)
14 days	102 (25.8%)
21 days	16 (04.1%)
30 days	09 (02.3%)
I do not know	98 (24.8%)
13. When should the first tube be changed?	
5-10 days^b^	143 (36.2%)
3 weeks	43 (10.9%)
1 month	53 (13.4%)
3 months	63 (15.9%)
I do not know	93 (23.5%)
14. Mandatory bedside tracheostomy equipment that should be maintained constantly^a^	
Tracheal dilator*	246 (62.3%)
Spare tracheostomy tube of a larger size	101 (25.6%)
Spare tracheostomy tube of the same size^b^	221 (55.9%)
Spare tracheostomy tube of a smaller size^b^	109 (27.6%)
Yankauer suction tip	208 (52.7%)
Endotracheal tube	147 (37.2%)
I do not know	58 (14.7%)
15. What is the first measure that should be taken for accidental decannulation?	
Reinsert the tube^b^	84 (21.3%)
Bag mask ventilation	156 (39.5%)
Jaw thrust	17 (04.3%)
Head tilt chin lift	58 (14.7%)
I do not know	80 (20.3%)
16. Which instrument will facilitate re-insertion of the tracheostomy tube?	
Laryngoscope	38 (09.6%)
Tracheal dilator^b^	181 (45.8%)
Obturator	58 (14.7%)
Suction catheter	19 (04.8%)
I do not know	99 (25.1%)
17. What is the first measure that should be taken for tube obstruction?	
Ask patient to cough	77 (19.5%)
Remove the tube	56 (14.2%)
Insert suction^b^	130 (32.9%)
Deflate the cuff	68 (17.2%)
I do not know	64 (16.2%)
18. Which of the following is a sign of tube obstruction?^a^	
Noisy breathing^b^	211 (53.4%)
Difficult breathing^b^	267 (67.6%)
Use of accessory muscles^b^	212 (53.7%)
Abdominal pain	69 (17.5%)
Drop in oxygen saturation^b^	283 (71.6%)
Difficulty passing suction catheter^b^	230 (58.2%)
I do not know	46 (11.6%)
19. What is the first measure that should be taken for tracheostomy bleeding?	
Inflate the cuff^b^	33 (08.4%)
Remove the tube	27 (06.8%)
Apply pressure dressing	172 (43.5%)
Start suctioning	87 (22.0%)
I do not know	76 (19.2%)
20. Which of the following signs indicate infection?^a^	
Fever^b^	366 (92.7%)
Granulation tissues^b^	126 (31.9%)
Tissue breakdown^b^	125 (31.6%)
Foul odor mucus^b^	252 (63.8%)
Increased respiratory rate^b^	184 (46.6%)
Swelling^b^	215 (54.4%)
I do not know	26 (06.6%)
21. Which of the following measures will help in reducing tracheostomy-related infections?^a^	
Systemic antibiotics	273 (69.1%)
Wet dressing^b^	107 (27.1%)
Swab if there is any secretion^b^	208 (52.7%)
Topical steroid for granulation tissues^b^	111 (28.1%)
I do not know	48 (12.2%)
**Total knowledge score (mean ± SD)**	**21.6 ± 9.11**
Level of knowledge	
Poor	232 (58.7%)
Moderate	154 (39.0%)
Good	9 (02.3%)

^a^Variables with multiple responses

^b^Indicates the correct answer

As presented in Table 3, higher knowledge scores were strongly associated with having a higher education level, such as a master’s degree or PhD (H = 17.756; *p* < 0.001), work experience in a tertiary hospital (H = 71.332; *p* < 0.001), working at a government institution (Z = 4.665; *p* < 0.001), caring for patients with tracheostomies (Z = 4.729; *p* < 0.001), and attending lectures or courses about managing patients with tracheostomies (Z = 3.313; *p* < 0.001). Conversely, lower knowledge scores were associated with nurses aged between 20 and 30 years (H = 22.189; *p* < 0.001) and those with < 5 years of experience (H = 40.827; *p* < 0.001).

**Table 3:** Differences in knowledge score according to socio-demographic characteristics of nurses (*s* = 348)

Factor	Knowledge score mean ± SD	Z/H value	*P*-value
Age group^a^			
20–30 years	18.9 ± 9.37	H = 22.189	< 0.001**
31–40 years	23.4 ± 8.87		
> 40 years	22.9 ± 7.57		
Sex^b^			
Male	20.9 ± 9.63	Z = 0.813	0.416
Female	21.8 ± 9.00		
Education level^a^			
Diploma	17.9 ± 9.79	H = 17.756	< 0.001**
Bachelor’s degree	22.2 ± 8.72		
Master’s degree or PhD	26.9 ± 8.45		
Years in practice^a^			
< 5 years	16.9 ± 9.01	H = 40.827	< 0.001**
5–10 years	23.4 ± 8.65		
> 10 years	23.7 ± 8.28		
Level of care provided by hospital^a^			
Primary	16.8 ± 8.69	H = 71.332	< 0.001**
Secondary	17.8 ± 7.87		
Tertiary	24.9 ± 8.28		
Type of institution^b^			
Government	22.5 ± 8.98	Z = 4.665	< 0.001**
Private	16.5 ± 8.27		
Where do you work in the hospital?^a^			
Medical ward	20.8 ± 10.7	H = 2.333	0.801
Surgical ward	22.0 ± 7.55		
ICU	22.2 ± 8.08		
OPD	21.7 ± 9.90		
OR	23.1 ± 7.87		
ER	20.6 ± 7.59		
Previously cared for tracheostomized patients^b^			
Yes	22.9 ± 8.17	Z = 4.729	< 0.001**
No	17.0 ± 10.8		
Attended course or lecture about managing patients with tracheostomy^b^			
Yes	22.8 ± 8.54	Z = 3.313	< 0.001**
No	19.4 ± 9.72		

*Abbreviations: OPD* Outpatient department, *ICU* Intensive care unit, *OR* Operating room, *ER* Emergency room

^a^*p*-value calculated using Kruskal–Wallis H-test

^b^*p*-value calculated using Mann–Whitney Z-test

^**^Significant at *p* < 0.05 level

## Discussion

This study aimed to evaluate nurses’ readiness to manage tracheostomy-related complications. Nurses’ readiness was measured by establishing their knowledge level and associated factors. Our analysis revealed that 58.7%, 39%, and 2.3% of nurses had poor, moderate, and good knowledge levels, respectively (mean ± standard deviation score: 21.6 ± 9.11 of 49 points). The results of several studies worldwide are consistent with our main finding demonstrating significant deficiency in tracheotomy care-related knowledge among nurses and their abilities to manage complications [13–15].

Our data revealed that nurses who worked in tertiary hospitals or government institutions with higher professional degrees (master’s and PhD) and previous experience with tracheostomized patients exhibited a readiness to manage tracheostomy-related care. These nurses had also attended courses and lectures on tracheostomy-related care. This result is contrary to the findings of Khanum et al. [13], who reported that nurses aged between 26 and 30 years had significantly higher knowledge scores, and no significant differences in qualifications and years of tracheostomy care among nurses were identified.

Confidence is a critical aspect in the management of tracheostomized patients. Our results demonstrated that almost half of the nurses had no previous experience with tracheostomy-related emergencies, and many lacked confidence in managing such cases. The need for educational programs and training to close gaps in knowledge is vital to achieve the optimal quality of care. Better knowledge has been observed among nurses in hospitals that invest in the implementation of educational content on tracheostomy care [16]. Sufficient knowledge of tracheostomy management and care is necessary, as it may translate into better responses in actual practice. Consistent with our reports, another study [17] reported that comfort with a tracheostomy was higher among nurses with at least 5 years of experience, continuous exposure, and close work with tracheostomized patients. This highlights the importance of training and exposure, which reflect nurses’ confidence in the management of tracheostomized patients in actual clinical practice.

Concerns about the gaps in knowledge and practices can be addressed through in-service continuous professional development for registered nurses working in all units, such as internal medicine, surgical wards, and intensive care units, which will help to identify the major factors contributing to these inadequacies [18, 19].

 We conjecture that the shortage of nurses is another critical issue contributing to this problem. A recent report in Saudi Arabia revealed that the number of nurses per 100,000 inhabitants was 5.5 in 2018, falling behind developed countries such as the United States (US), UK, and Australia [18]. Several reports have indicated that the lack of adequate nursing staff negatively impacted patient care and the nurses themselves, resulting in burnout, anxiety, and lack of adequate time for updating their medical knowledge [19, 20].

Despite our significant results, there are some limitations to our study, particularly the risk of response bias. Additionally, the parameters used in the questionnaire have not been validated. Future studies should include more variables, such as working conditions, psychological factors, and burnout measurement.

### Recommendations for future research

Future research should further identify the workload, social, and psychological factors contributing to the knowledge gap concerning tracheostomy-related practices among nurses in Saudi Arabia.

### Clinical implications for nursing managers and policymakers

A potential factor contributing to the current issue is the shortage of nurses in Saudi Arabia and limited nursing school capacity. Data from 2015 revealed that the number of nursing school graduates per 100,000 people was 11, which is low compared with that in other countries, such as the UK, US, and Australia (68, 62, and 78, respectively). Accordingly, universities should increase their capacity to meet the large demand for education and to improve the curriculum.

## Conclusions

Nurses are the frontline caregivers for tracheostomized patients and must therefore maintain the appropriate skill set and self-efficacy to provide safe and effective care. A lack of such skills and knowledge can be life-threatening. The current findings have significant clinical applications, as we identified deficiencies in nursing knowledge that align with the observed gaps in tracheostomy patient care. This highlights the need to implement further education and training. Nurses who received tracheostomy training had significantly higher knowledge scores. This is essential to nursing education and should also constitute part of a hospital's broader initiatives to improve and to maintain high-quality patient care and satisfaction. Our findings suggest that implementing targeted training programs for tracheostomy care may be an effective training strategy to ensure standardized nursing competencies for specialized care.

## Supplementary Information

**Additional file 1.** Supplementary Material.

## Data Availability

The datasets used and/or analyzed during the current study are available from the corresponding author upon reasonable request.

## Abbreviations

OPD: Outpatient department
ICU: Intensive care unit
OR: Operating room
ER: Emergency room

## References

[1] Monteiro S, de Farias TP, de Camargo Millen M, Locio RV, de Farias T (2018). The history of tracheostomy. tracheostomy.

[2] Morris LL, Whitmer A, McIntosh E (2013). Tracheostomy care and complications in the intensive care unit. Crit Care Nurse.

[3] Farida JP, Lawrence LA, Svider PF, Shkoukani MA, Zuliani GF, Folbe AJ (2016). Protecting the airway and the physician: aspects of litigation arising from tracheotomy. Head Neck.

[4] Wilkinson K, Freeth H, Kelly K (2015). 'On the Right Trach?' A review of the care received by patients who undergo tracheostomy. Br J Hosp Med (Lond).

[5] Sodhi K, Shrivastava A, Singla MK (2014). Implications of dedicated tracheostomy care nurse program on outcomes. J Anesth.

[6] Veelo DP, Schultz MJ, Phoa KYN, Dongelmans DA, Binnekade JM, Spronk PE (2008). Management of tracheostomy: a survey of Dutch intensive care units. Respir Care.

[7] Krejcie RV, Morgan DW (1970). Determining sample size for research activities. Educ Psychol Meas.

[8] Ministry of Health. Statistical Yearbook. Health Mo ed. Saudi Arabia: Ministry of Health; 2018. https://www.moh.gov.sa/en/Ministry/Statistics/book/Documents/book-Statistics.pdf.

[9] Yelverton JC, Nguyen JH, Wan W, Kenerson MC, Schuman TA (2015). Effectiveness of a standardized education process for tracheostomy care. Laryngoscope.

[10] Dawson D (2014). Essential principles: tracheostomy care in the adult patient. Nurs Crit Care.

[11] St John RE, Malen JF (2004). Contemporary issues in adult tracheostomy management. Crit Care Nurs Clin North Am.

[12] Flaherty C (2020). Tracheostomy care: the role of the nurse before, during and after insertion. Nurs Stand.

[13] Khanum T, Zia S, Khan T, Kamal S, Khoso MN, Alvi J (2022). Assessment of knowledge regarding tracheostomy care and management of early complications among healthcare professionals. Braz J Otorhinolaryngol.

[14] Qadir MB (2018). A study to assess the impact of structured teaching programme on knowledge regarding tracheostomy care among staff nurses working in selected hospital of Kashmir. Int J Nurs Res.

[15] Gaterega T, Mwiseneza MJ, Chironda G (2021). Nurses knowledge and practices regarding tracheostomy care at a selected referral hospital in Rwanda – a descriptive cross-sectional study. Int J Afr Nurs Sci.

[16] Alnemare AK (2020). Nurses training and confidence in management of tracheostomy patients in a community hospital in Saudi Arabia. J Res Med Dent Sci.

[17] Dorton LH, Lintzenich CR, Evans AK (2014). Simulation model for tracheotomy education for primary health-care providers. Ann Otol Rhinol Laryngol.

[18] McDonough K, Crimlisk J, Nicholas P, Cabral H, Quinn EK, Jalisi S (2016). Standardizing nurse training strategies to improve knowledge and self-efficacy with tracheostomy and laryngectomy care. Appl Nurs Res.

[19] Machitidze M (2022). Impact of the nurses education and shortage on the patients care outcomes-literature review. Am J Biomed Sci Res.

[20] Akroute AR, Brinchmann BS, Hovland A, Fredriksen SD (2022). ICU nurses’ lived experience of caring for adult patients with a tracheostomy in ICU: a phenomenological-hermeneutic study. BMC Nurs.

