# Supplementary material for: Assessment of the ability, perception, and readiness of nurses to manage tracheostomy-related complications in Riyadh City: a cross-sectional study
Source: BMC Nurs. 2022 Nov 23;21:320. doi: 10.1186/s12912-022-01101-y (PMC9682812; doi:10.1186/s12912-022-01101-y)
Supplement: Supplementary file 1 — Additional file 1. Supplementary Material. [file 12912_2022_1101_MOESM1_ESM.docx]

**Supplementary Material**

**#Informed consent:**

| We are a research group from Imam Mohammad Ibn Saud Islamic University - College of Medicine. We invite you to participate in this survey.  If you agree to participate, please be aware that the survey will take a maximum of 10 min. All answers will be handled with privacy and discretion and will only be accessible by the research team. Your participation is not mandatory, and you have the right not to participate or to withdraw at any time.  Thank you for your time and cooperation.  Main Author:  Dr. Fahad Al Otaibi  Contact information: fzalotaibi@imamu.edu.sa  **Do you agree to participate?**  · Yes  · No |
| --- |

**1. Demographic data:**

1.1 Age, in years (20–30 / 31–40 / 41–50 / 51–60)

1.2 Sex (male / female)

1.3 Educational and qualification level (diploma / bachelor's degree / master’s / PhD)

1.4 Years in practice (< 5 / 5–10 / > 10)

**2. Practice setting and experience:**

2.1 Level of care provided by hospital: (primary / secondary / tertiary)

2.2 Where do you work in the hospital? (medical floor / surgical floor / ICU / OPD / OR / ER)

2.3 Have you taken care of tracheostomized patients before? (Yes / No)

2.4 Have you ever taken a course or lecture about managing patients with a tracheostomy? (Yes / No)

**3. Basic knowledge of tracheostomy tube components and indications:**

3.1. Which of the following is an indication to perform tracheostomy?*(you can choose more than one) (airway obstruction / prolonged mechanical ventilation / infections / neuromuscular disease / inability to intubate / congenital anomaly / neck trauma / neoplasm / bilateral vocal cord paralysis / facial fracture / prophylaxis in head and neck procedures)

3.2. How frequently should the inner cannula be checked and cleaned? (once a day / twice a day / every 2 days / once a week / I do not know)

3.3. Which of the following is a component of the tracheostomy care kit? (inner cannula / obturator / plug / hydrogen peroxide )

3.4. What are the characteristics of an ideal tracheostomy stoma? (clean and dry / wet / full of granulation tissues / I do not know)

3.5. What is the ideal cuff pressure? (< 25 cm H2O / > 26 cm H2O / < 35 cm H2O / > 35 cm H2O)

3.6. When should the cuff be deflated? (when the patient is off the MV / cuff should always be kept inflated / I do not know)

3.7. Do patients with a tracheostomy require ‘humidification’? (Yes / No / I do not know) 3.8. How frequently should ‘suctioning’ be performed? (only when indicated / routinely every 2 h / routinely every 4 h / routinely each shift)

3.9. Which of the following is an indication for suctioning?* (you can choose more than one) (audible or visual signs of secretions in the tube / signs of respiratory distress / blocked or partially blocked tube / vomiting / desaturation on pulse oximetry )

3.10. What is the appropriate suctioning pressure? (80–120 mmHg / 120–150 mmHg / 50–70 mmHg / I do not know )

3.11. What is the appropriate length of the suction tube to length of the inner tracheostomy cannula? (same length / longer / shorter / I do not know)

3.12. When should the sutures be removed after surgical insertion? (day 5–7 / day 20 / day 30)

3.13. When should the first tube be changed? (day 5–10 / 3 weeks / 1 month / 3 months)

**4. Management of tracheostomy complications:**

4.1. Which of the following is mandatory bedside tracheostomy equipment that should be kept at all times? (you can choose more than one)* (tracheal dilator / spare tracheostomy tube of a larger size / spare tracheostomy tube of the same size / spare tracheostomy tube of a smaller size / Yankauer suction tip / endotracheal tube)

Accidental decannulation:

4.2. Have you dealt with this complication before? (Yes / No)

4.3. What is the first measure to take? (reinsert the tube / bag mask ventilation / jaw thrust / head tilt chin lift)

4.4. Which instrument will facilitate reinsertion of the tracheostomy tube? (laryngoscope / tracheal dilator / obturator / suction catheter)

Tube obstruction:

4.5. Have you dealt with this complication before? (Yes / No)

4.6. What is the first measure to take? (ask the patient to cough / remove the tube / insert suction / deflate the cuff)

4.7. Which of the following is a sign of tube obstruction (you can choose more than one)* (noisy breathing / difficulty breathing / use of accessory muscles / abdominal pain / drop in oxygen saturation / difficulty passing suction catheter)

Tracheostomy bleeding:

4.8. Have you dealt with this complication before? (Yes / No)

4.9. What is the first measure to take? (inflate the cuff / remove the tube / apply pressure dressing / start suctioning)

Infections:

4.10. Which of the following signs indicate infection? * (fever / granulation tissues / tissue breakdown / mucopurulent discharge / foul odor mucus / increased RR / stoma site bleeding / thick mucus / swelling)

4.11. Which of the following measures will help in reducing tracheostomy-related infections?* (systemic antibiotics / wet dressing / swab if there is any secretion / topical steroid for granulation tissues)

* participant can choose more than one answer
